# Pregnane-X-Receptor Controls Hepatic Glucuronidation During Pregnancy and Neonatal Development in Humanized *UGT1* Mice

**DOI:** 10.1002/hep.25671

**Published:** 2012-06-11

**Authors:** Shujuan Chen, Mei-Fei Yueh, Ronald M Evans, Robert H Tukey

**Affiliations:** 1 Department of Pharmacology, Laboratory of Environmental Toxicology, University of CaliforniaSan Diego, La Jolla, CA; 2 Howard Hughes Medical Institute and Gene Expression Laboratory, Salk Institute of Biological StudiesLa Jolla, CA; 3 Department of Chemistry & Biochemistry, Laboratory of Environmental Toxicology, University of CaliforniaSan Diego, La Jolla, CA

## Abstract

In humanized UDP glucuronosyltransferase-1 (*hUGT1*) mice that express the entire *UGT1* locus, the maternal hepatic *UGT1A* genes are dramatically induced 12-14 days after conception. Steroid induction of the *UGT1A1* gene indicates that xenobiotic sensors, such as the pregnane X receptor (PXR) and constitutive androstane receptor (CAR), may underlie the induction process. In contrast, neonatal *hUGT1* mice display severe hyperbilirubinemia, with limited expression of the *UGT1A* genes. This study identifies PXR as both a positive and negative regulator of the *UGT1A1* gene. Pregnancy hormones, in particular the glucocorticoids, target PXR as a positive regulator of human glucuronidation. Employing reverse genetics, where PXR has been genetically deleted, *hUGT1/Pxr*^−/−^ mice show limited induction of the liver *UGT1A* genes during pregnancy, whereas the exact opposite occurs in newborn mice. Neonatal *hUGT1* mice show delayed expression of hepatic UGT1A1 and are severely hyperbilirubinemic. However, in *hUGT1/Pxr*^−/−^ mice, hyperbilirubinemia is greatly reduced due to induction of hepatic UGT1A1. Thus, PXR serves to repress *UGT1A1* gene expression during development. Transcriptional silencing of the *UGT1A1* gene was relieved in neonatal *hUGT1* hepatocytes through interruption of PXR by small interfering RNA. *Conclusion*: PXR is a key regulator of pregnancy induced glucuronidation capacity in addition to modulating the severity of neonatal jaundice. (Hepatology 2012;56:658–667)

Pregnancy results in dramatic surges in hormones during gestational development, with increases in steroids such as estrogens, progestins, and the glucocorticoids. These steroids have been linked to the control and regulation of xenobiotic drug metabolism, primarily through activation of the nuclear xenobiotic receptors such as the pregnane X receptor (PXR)^1^-^3^ and the constitutive androstane receptor (CAR).^4^, ^5^ PXR and CAR have been classified as steroid and xenobiotic sensors, so it can be anticipated that significant fluctuations in the pregnancy hormones will modulate transcriptional control of target genes such as those involved in xenobiotic or drug metabolism.

Clinical findings indicate that steroid fluctuations lead to changes in xenobiotic glucuronidation during pregnancy. For example, circulating unconjugated bilirubin is cleared from the circulation solely through UDP glucuronosyltransferase 1A1 (UGT1A1) metabolism.^6^ During pregnancy, total serum bilirubin (TSB) levels are lower in women,^7^ indicating that bilirubin metabolism is accelerated through induced UGT1A1. Labetalol, an antihypertensive agent, which is metabolized primarily by UGT1A1 glucuronidation, shows increased clearance in the second and third trimesters of pregnancy compared to the postpartum period.^8^ Lamotrigine, an antiepileptic agent metabolized by UGT1A3 and UGT1A4, has a 50% decreased elimination half-life with an increased clearance of over 200% during pregnancy, leading to a closely correlated higher incidence of epileptic seizures.^9^ Labetalol and lamotrigine clearance during pregnancy indicates that UGT1A1, UGT1A3, and UGT1A4 are induced and clearance is accelerated. It has been suggested that up-regulation of UGT1A4 during pregnancy may be mediated by 17β-estradiol and the estrogen receptor alpha (ERα).^10^ Clearly, hormonal sensors during pregnancy are leading to induction of human glucuronidation capacity.

The exact opposite is occurring during neonatal development, which is evident by the very high incidence of hyperbilirubinemia in newborn children. Because bilirubin is metabolized exclusively by UGT1A1,^6^ hyperbilirubinemia develops from the inability of liver glucuronidation to match the early rise in serum bilirubin that forms from the abundance of red blood cells needed to carry oxygen. Senescence of the erythrocytes leads to an accumulation of hemoglobin that is rapidly metabolized into bilirubin and released into the circulation, where it is transported to the liver for excretion following UGT1A1-dependent glucuronidation. Jaundice is directly linked to inadequate glucuronidation of serum bilirubin stemming from reduced expression of liver UGT1A1.^11^-^13^ It is unclear if the reduced expression of UGT1A1 in neonates is a controlled event through transcriptional silencing or simply a result of limited epigenetic factors that are eventually produced to positively regulate the *UGT1A1* gene in a developmental fashion.

In this report it will be demonstrated that PXR is linked to both pregnancy-induced expression of the *UGT1* locus as well as repression of the *UGT1A1* gene in neonatal development. These findings were generated through the development of humanized *UGT1* (*hUGT1*) mice,^14^, ^15^ which express the entire human *UGT1* locus in a murine *Ugt1*-null background.^16^ Taking advantage of the power of reverse genetics, it will be shown that PXR plays a crucial role in pregnancy-induced glucuronidation in addition to the early development of hyperbilirubinemia in neonatal *hUGT1* mice.

## Materials and Methods

### Animals

The generation of Tg(UGT1^A1*1^)Ugt1^−/−^ (hUGT1*1) and Tg(UGT1^A1*28^)Ugt1^−/−^ (hUGT1*28) mice has been reported.^15^*Pxr*^−/−^ mice were generated as described^17^ and *Car*^−/−^ mice were generously provided by Dr. Masahiko Negishi (NIEHS). All genetically modified strains were bred for over five generations with C57BL/6 wildtype mice before inbreeding. To generate *hUGT1/Pxr*^−/−^ mice, hUGT1*1 mice were crossed with *Pxr*^−/−^ mice, producing Tg(UGT1^A1*1^)Ugt1^+/−^Pxr^+/−^ mice. These mice were backcrossed in brother/sister matings to generate Tg(UGT1^A1*1^)Ugt^−/−^Pxr^−/−^ (hUGT1*1/Pxr^−/−^) mice. The same breeding strategy was used to generate hUGT1*1/Car^−/−^ mice.

### Primary Hepatocyte Isolation and PXR-Targeted Specific Small Interfering RNA (siRNA) Regulation

Hepatocytes were isolated as described.^14^ The hepatocytes were then cultured in 6-well collagen-treated plates (Discovery Labware, Bedford, MA) in 2 mL of Dulbecco's modified Eagle's medium (DMEM) containing penicillin/streptomycin and supplemented with 10% fetal bovine serum. siRNA duplexes specific for mouse PXR were provided by Bioneer (Alameda, CA) and Santa Cruz Biotechnology. Four hours after primary hepatocytes were isolated from 14-day-old hUGT1*1 mice, cells were transfected in the presence of 20 nM of either siRNA or control RNA with Lipofectamine 2000 (Invitrogen) in a final volume of 0.5 mL of OPTI-MEM. After 5 hours cells were changed with fresh medium supplemented with 10% fetal bovine serum and penicillin-streptomycin. Forty-eight hours later, cells were used for RNA extraction. Reverse transcription (RT) and real-time polymerase chain reaction (Q-PCR) were carried out to examine gene expression levels of mouse *Pxr*, human *UGT1A1* and mouse *Cyp3a11*.

### Immunoblot Analysis and Real-Time PCR

Mice were sacrificed and livers were perfused with ice-cold 1.15% KCL and microsomes prepared as outlined.^14^ All western blots were performed using NuPAGE BisTris-polyacrylamide gels as described.^15^ For real-time quantitative Q-PCR analysis, ≍100 mg of liver tissue was homogenized into 1 mL of TRIzol and RNA prepared. Using iScript Reverse Transcriptase (BioRad), 1 μg of total RNA was used for the generation of complementary DNA (cDNA) as outlined by the manufacturer in a total volume of 20 μL. Following synthesis of cDNA, 2 μL was used in real-time PCR conducted with a QuantiTect SYBR GreenPCR kit (Qiagen, Valencia, CA) using a MX4000 Multiplex Q-PCR (Stratagene, La Jolla, CA) programmed to take three fluorescence data points at the end of each annealing plateau. All PCR reactions were performed in triplicate as outlined.^14^*Ct* values were normalized to mouse cyclophilin (CPH). The specific primers used to quantitate the respective gene transcripts^18^ are listed in Supporting Table 1.

### Chromatin Immunoprecipitation (CHIP)

CHIP analysis was performed using the modified protocol based on the EZ-CHIP kit (Millipore). Liver tissue (100 mg) was minced and cross-linked in DMEM (Invitrogen) containing 1% formaldehyde. The procedures for cell lysis and sonication to shear DNA were followed according to the manufacturer's protocol (EZ-CHIP kit, Millipore). One mL of cell extract was precleared by incubation with 60 μL of protein A Agarose/Salmon sperm DNA overnight at 4°C. The cleared cellular extract was incubated with anti-PXR antibody (Santa Cruz, sc-25381) for 2 hours at 4°C. Following precipitation with protein A agarose, the antibody-chromatin complex was then washed as outlined.^19^ The protein-DNA complexes were eluted in 200 μL elution buffer and DNA was then reverse cross-linked and released from the complex as indicated in the EZ-CHIP instructions. Following the DNA purification with spin columns (Qiagen), the purified DNA was further analyzed by PCR with a pair of primers (forward 5′-TTGTGGGGCAATACACTAGTA-3′, reverse 5′-GTCCGGGTTTCAGGTTATGTA-3′) for the amplification of the *UGT1A1* promoter region containing the PXR binding site.^3^

## Results

### Expression of the Human *UGT1A* Genes in TgUGT1 Mice During Pregnancy

**Fig. 1:**
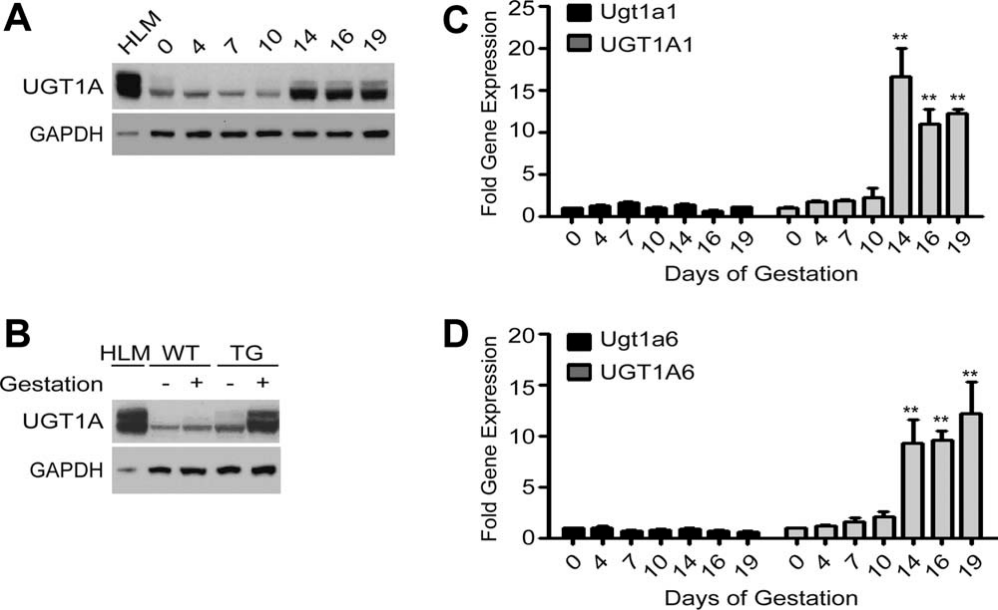
Induction of the *UGT1* locus in *TgUGT1* mice. Age-matched female *TgUGT1* mice were mated with wildtype mice. The following morning, female mice with the presence of a vaginal plug were removed, housed separately, and timed as gestation day 1. Pregnant mice were sacrificed at gestation days (GD) 4, 7, 10, 14, 16, and 19. Samples from at least three nonpregnant female *TgUGT1* mice were used as controls. Liver microsomes and RNA from the nonpregnant control and pregnant mice were prepared. (A) Immunoblot detection of UGT1A proteins. Liver microsomes prepared from pregnant *TgUGT1* mice at progressive stages of pregnancy were analyzed on 10% sodium dodecyl sulfate-polyacrylamide gel electrophoresis (SDS-PAGE) and the UGT1A proteins detected on immunoblots by using an anti-UGT1A antibody and anti-GAPDH antibody (Santa Cruz Biotechnology). Human liver microsomes (HLM) were used as a positive control. (B) Immunoblot of UGT1A proteins from liver microsomes prepared from female wildtype and *TgUGT1* mice that were pregnant for 16 days. Control samples were prepared from nonpregnant wildtype and *TgUGT1* mice. (C) Total RNA was isolated from liver samples taken from the pregnant *TgUGT1* mice and used in RT and Q-PCR analysis to examine murine UGT1A1 RNA (Ugt1a1) expression and human UGT1A1 RNA expression. Student's *t* test was used to evaluate the statistical significance (**P < 0.01). (D) The same RNA samples were used to quantitate murine *Ugt1a6* and human *UGT1A6* gene expression (**P < 0.01, *t* test).

Heterozygous female TgUGT1*28 mice were mated with wildtype mice and the presence of the vaginal plug in the morning was set as gestation day 1 (GD1). Starting at GD4, gravid mice were sacrificed at various times during pregnancy and liver microsomes used for western blot analysis where the UGT1A proteins were identified with a human anti-UGT1A antibody.^20^ Total UGT1A protein expression was induced significantly by the end of the second trimester (GD14), and remained at a high level of expression throughout the entire third trimester (Fig. 1A). This anti-UGT1A antibody also detects murine UGT1A proteins,^16^ but with wildtype mice as controls, no induction of murine UGT1A proteins during pregnancy was observed (Fig. 1B).

Two of the UGTs that are highly conserved in function between mice and humans are UGT1A1 and UGT1A6. Expression of the murine *Ugt1a1* and *Ugt1a6* genes along with the human *UGT1A1* and *UGT1A6* genes were evaluated by RT and Q-PCR analysis using species specific primers for each of these genes. Throughout pregnancy, we observed no induction of murine *Ugt1a1* or *Ugt1a6* gene expression (Fig. 1C,D), findings that correlated with the lack of wildtype UGT1A protein expression. Consistent with induction of UGT1A protein in TgUGT1*28 mice during pregnancy, *UGT1A1* and *UGT1A6* gene expression is induced at GD14, with the induction being sustained throughout the remainder of the gestational period. Introduction of the human *UGT1* locus and the *UGT1A* genes in TgUGT1*28 mice is regulated throughout pregnancy in a pattern that is not replicated by the murine *Ugt1* locus. Emerging principles of regulatory evolution strongly favor genetic diversity in *cis*-regulatory DNA and not *trans*-regulation of gene expression to explain interspecies differences in gene expression.^21^, ^22^ The differences in transcriptional regulation between the murine and human *UGT1* locus during pregnancy may be credited to important genetic differences in the regulatory regions of these genes.

### Induction of the *UGT1* Locus in Humanized *UGT1* Mice

**Fig. 2:**
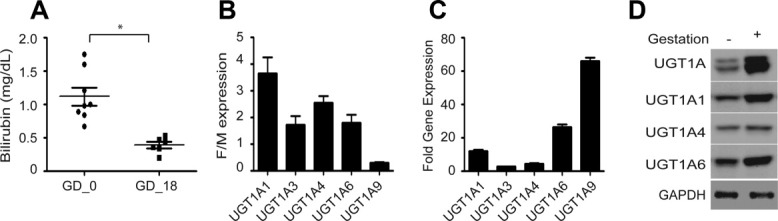
Regulation of the *UGT1* locus during pregnancy. (A) Serum bilirubin levels in female hUGT1*28 mice were determined before pregnancy and at GD18 (*P < 0.05, Student's *t* test). (B) Quantitation of liver *UGT1A* gene expression by RT and Q-PCR in adult female and male mice. The relative values are expressed as a ratio value comparing female to male expression. (C) Quantitation of liver *UGT1A* gene expression in hUGT1*1 mice pregnant for 16 days. Total liver RNA was prepared and used in RT and Q-PCR analysis with specific *UGT1A* gene oligonucleotides. Fold induction was calculated relative to those values obtained in nonpregnant mice. (D) Immunoblot analysis of human UGT expression in nonpregnant and pregnant hUGT1*1 mice. Microsomes were prepared from nonpregnant and pregnant hUGT1*1 mice and subjected to 4%-12% polyacrylamide gel electrophoresis. Following blotting, protein expression was determined using a UGT1A antibody, or isozyme specific UGT1A1, UGT1A4, and UGT1A6 antibodies.

We generated hUGT1*1 and hUGT1*28 mice, which differ predominantly in expression levels of UGT1A1 in adult liver.^15^ Adult hUGT1*28 mice are hyperbilirubinemic, with TSB levels that average 1 mg/dL. During pregnancy and late gestation, the TSB levels in hUGT1*28 mice averaged 0.4 mg/dL, over 50% lower than in nonpregnant mice (Fig. 2A). The reduction in TSB can be accounted for by elevated levels of liver UGT1A1.

In hUGT1*1 mice, expression of *UGT1A1, -1A3, -1A4*, and -*1A6* are 2 to 4-fold greater in female liver than male liver (Fig. 2B). The sole exception to the female dominance is *UGT1A9*, which has minimal expression in female liver. Examination of the fold increase in gene expression of each of the *UGT1A* genes shows that *UGT1A9* expression increases to nearly 70-fold over nonpregnant values (Fig. 2C). This large increase in *UGT1A9* expression accounted for by fold induction during pregnancy results in part from the very low basal levels observed in nonpregnant female mice. Along with *UGT1A9* gene expression, *UGT1A1* and *UGT1A6* gene expression are found to dominant the induction process during pregnancy. These increases are also reflected in microsomal protein abundance as determined by western blot analysis using isoform specific antibodies (Fig. 2D).

### Pregnancy Steroids and Induction of UGT1A1

**Fig. 3:**
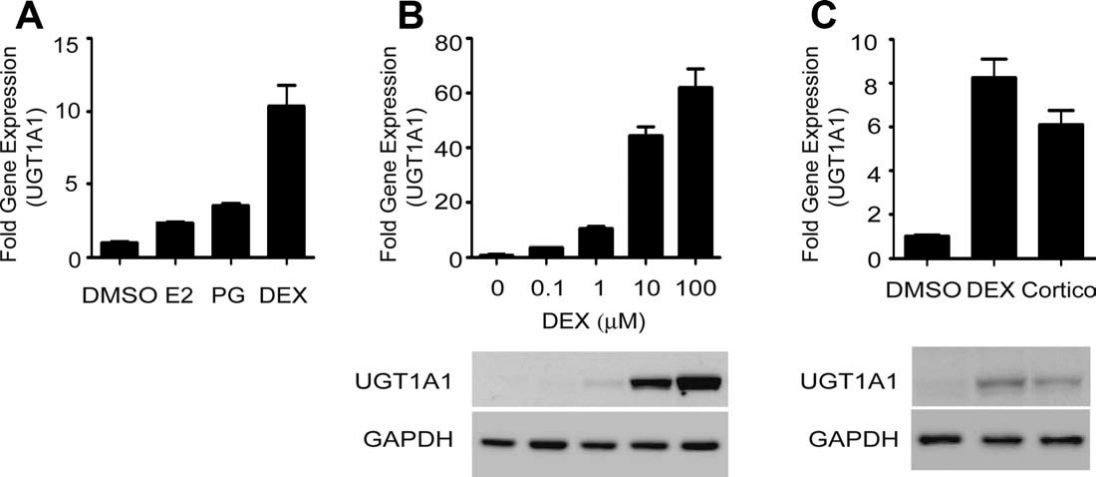
Induction of *UGT1A1* in primary hepatocytes by pregnancy-related hormones. (A) Primary hepatocytes were isolated from hUGT1*1 mice and treated with either estrogen (20 μg/mL), progesterone (50 μg/mL), or DEX (1 μM). After 24 hours RNA was isolated and *UGT1A1* gene expression was determined by RT and Q-PCR. Ct values from RT and Q-PCR were normalized to the housekeeping gene CPH and calculated as fold induction over the expression levels of cells treated with vehicle control. (B) Primary hepatocytes were exposed to different concentrations of DEX. After 24 hours RNA was isolated and *UGT1A1* gene expression monitored by RT and Q-PCR. A sample of total cell extract was also prepared and used for immunodetection of UGT1A1 and GAPDH by western blot analysis. (C) Isolated hepatocytes were exposed to DEX (1 μM) or corticosterone (20 μM) for 24 hours, followed by RT and Q-PCR analysis and western blot analysis to detect human UGT1A1 expression, and GAPDH blotting was used as loading control.

We hypothesized that hormone surges in late pregnancy play an important role in the gestational regulation of the human *UGT1* locus. To examine if selective steroids are capable of regulating the *UGT1A* genes, primary hepatocytes from hUGT1*1 mice were isolated, placed in culture, and exposed to 17β-estradiol, progesterone, or the synthetic glucocorticoid, dexamethasone (DEX). We measured induction of the *UGT1A1* gene because the other *UGT1A* genes are refractive to expression in hepatocytes in culture. Progesterone (50 μg/mL) and estradiol (20 μg/mL) exposure for 24 hours resulted in a minimal 2 to 3-fold induction of the *UGT1A1* gene (Fig. 3A). These concentrations were found to be optimal for *UGT1A1* induction. The synthetic glucocorticoid DEX (10 μM) induced *UGT1A1* gene expression up to 60-fold (Fig. 3B). This induction of gene expression and UGT1A1 induction is dose-dependent, with transcriptional induction of the *UGT1A1* gene in hepatocytes being substantial at 0.1 μM. Because corticosterone is the primary form of glucocorticoids in mouse, 20 μM of corticosterone was used to treat freshly isolated hepatocytes (Fig. 3C). Twenty-four hours after exposure, increased UGT1A1 protein levels were detected in whole cell lysates by western blot analysis, indicating that glucocorticoids play an important role in *UGT1A* gene expression.

### Xenobiotic Receptors and Induction of the *UGT1* Locus During Pregnancy

The progestins, corticosterone, and estradiols are low-affinity substrates for PXR^23^-^25^ and 17β-estradiol has been shown to activate CAR.^4^ Thus, experiments were conducted to examine precisely the role of PXR and CAR toward induction of the *UGT1* locus during pregnancy.

**Fig. 4:**
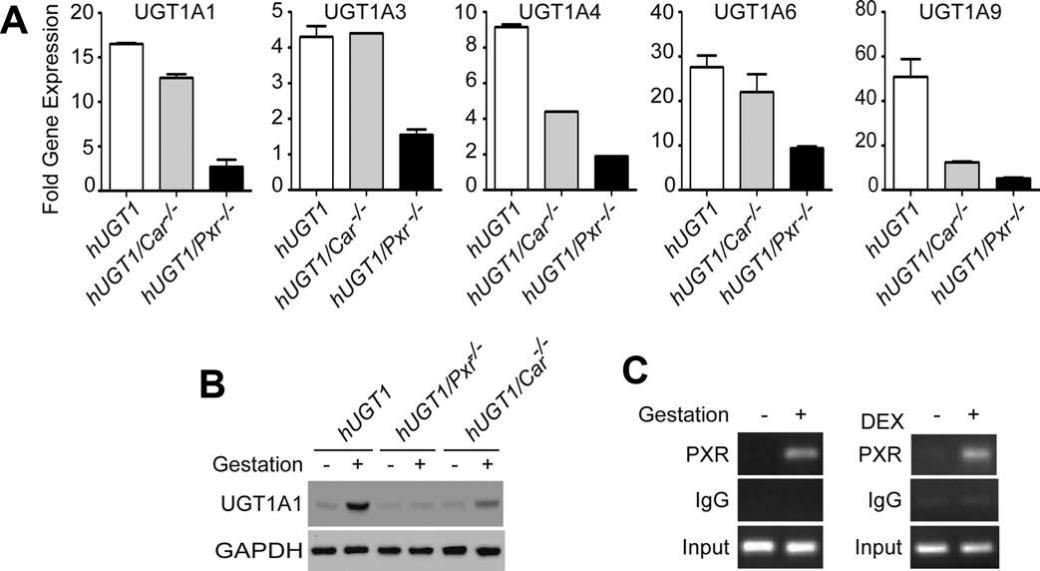
The impact of *PXR* and *CAR* deletion on gestational regulation of the human *UGT1* locus in liver tissue. Humanized *UGT1/Pxr*^−/−^ or *hUGT1/Car*^−/−^ mice were obtained by backcrossing hUGT1*1 mice with *Pxr*^−/−^ mice or *Car^−/−^* mice. Female hUGT1*1, hUGT1/Pxr^−/−^, and *hUGT1/Car*^−/−^ mice at 8 weeks old were used for timed pregnancy experiments. Age-matched nonpregnant female mice from each strain were used as controls. Mice were sacrificed at GD16. (A) RNA was isolated from pooled liver samples followed by RT and Q-PCR analysis. Primers specific for human UGT1A1, UGT1A3, UGT1A4, UGT1A6, and UGT1A9 gene products were used (Supporting Table 1). Q-PCR results from pregnant mice were normalized by the housekeeping gene CPH and described as fold of induction over the nonpregnant control. (B) Western blot analysis using liver microsomes and anti-UGT1A1 and anti-GAPDH antibodies. (C) CHIP analysis of PXR associated with the human *UGT1A1* gene in nonpregnant and 16 day pregnant *hUGT1* mice, and DEX-treated adult females.

To undertake these studies, hUGT1*1 mice were crossed with *Car*-null mice to create hUGT1*1/Car^−/−^ mice. On GD16, liver samples from hUGT1*1/Car^−/−^ mice were processed for total RNA along with microsomal extracts. RT and Q-PCR analysis for liver *UGT1A* gene products were conducted with specific oligonucleotide primers for each gene (Fig. 4A). In hUGT1*1/Car^−/−^ mice, gestational induction of the *UGT1A1, -1A3*, and -*1A6* genes was found to be similar to that observed in hUGT1*1 mice. Using western blot analysis to examine UGT1A1 expression in liver microsomes, UGT1A1 was induced during pregnancy in hUGT1*1 and hUGT1*1/Car^−/−^ mice (Fig. 4B). However, CAR does play a role in the induction of UGT1A4 and UGT1A9. We observed approximately a 50% reduction in the 8-fold increase in UGT1A4 RNA accumulation observed in hUGT1*1 mice. When UGT1A9 expression was analyzed, the robust induction in hUGT1*1 pregnant mice was reduced over 75% during pregnancy in hUGT1*1/Car^−/−^ mice, indicating an important role for CAR in the induction of the *UGT1A9* gene (Fig. 4A).

When hUGT1*1 mice are placed into a *Pxr*-null background, there was substantially reduced induction of each of the *UGT1A* genes during pregnancy when compared to expression in hUGT1*1 mice (Fig. 4A). The *UGT1A1* gene, robustly induced around 15-fold in hUGT1*1 and hUGT1*1/Car^−/−^ mice during pregnancy, displays reduced expression at GD16 in hUGT1*1/Pxr^−/−^ mice. A similar pattern of expression was observed when UGT1A1 was detected by western blot analysis, showing little expression in hUGT1*1/Pxr^−/−^ mice (Fig. 4B). An important role for PXR binding to the *UGT1A1* gene during pregnancy was reinforced when we examined PXR binding by CHIP analysis to the PXR binding site that flanks the *UGT1A1* promoter.^3^ PXR is activated during pregnancy and binds to the *UGT1A1* gene as demonstrated by CHIP analysis (Fig. 4C), indicating that endogenous ligands are participating in regulation of this gene. Coupled with CHIP analysis showing induced binding of PXR to the *UGT1A1* gene following DEX treatment (Fig. 4C) along with previous experiments demonstrating that PXR binding to this region of the *UGT1A1* gene stimulates transactivation of the promoter,^3^ these findings confirm that induction of UGT1A1 is closely linked to activation of PXR during pregnancy.

### Glucocorticoids Induce the *UGT1* Locus in a PXR-Dependent Fashion

**Fig. 5:**
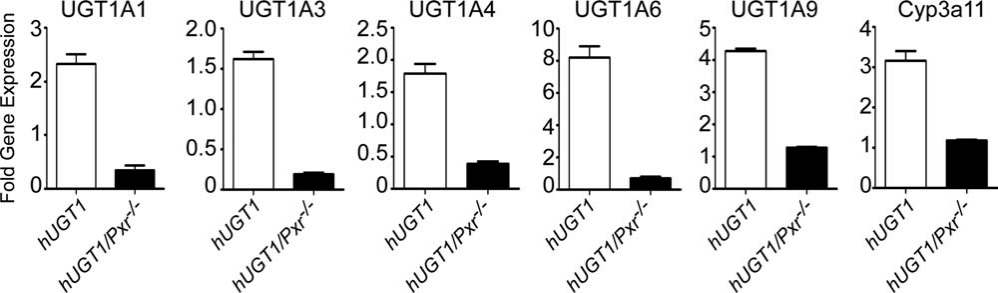
Induction of the *UGT1* locus by DEX in adult hUGT1*1 and *hUGT1/Pxr^−/−^* mice. Adult hUGT1*1 and *hUGT1/Pxr*^−/−^ mice were treated with DEX by intraperitoneal injection for 4 consecutive days at 20 mg/kg per dose. Nontreated mice received solvent and were used as controls. Twenty-four hours after the last dose, liver RNA was prepared and RT and Q-PCR analysis of human *UGT1A1, UGT1A3, UGT1A4, UGT1A6, UGT1A9*, and murine *Cyp3a11* gene expression was performed. Fold induction reflects the change in gene expression between solvent-treated and DEX-treated mice.

Because regulation of the *UGT1* locus during pregnancy is linked to PXR, we examined if the genes associated with the *UGT1* locus in humanized mice could be activated in a PXR-dependent fashion by glucocorticoids. We treated 8-week-old hUGT1*1 and hUGT1*1/Pxr^−/−^ female mice by the intraperitoneal route with 20 mg/kg DEX for 4 days and measured *UGT1A* gene expression in liver 48 hours after treatment (Fig. 5). Each of the five *UGT1A* genes expressed in liver was induced in hUGT1*1 mice. Although activation of the glucocorticoid receptor by DEX has been shown to activate *UGT1A1* reporter gene constructs in HepG2 cells,^26^ DEX treatment had no effect on induction of *UGT1A1* in hUGT1*1/Pxr^−/−^ mice, indicating that induction of the *UGT1A* genes by glucocorticoids is facilitated solely by activation of the PXR *in vivo*.

**Fig. 6:**
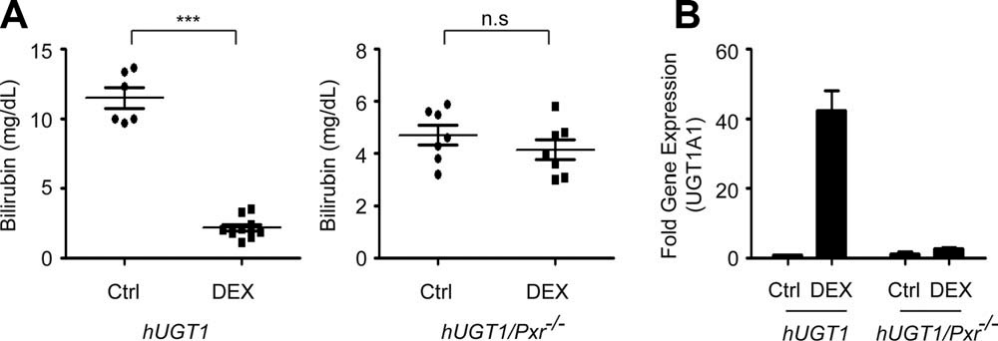
Induction of *UGT1A1* by DEX in neonatal hUGT1*1 and *hUGT1/Pxr^−/−^* mice. Humanized UGT1*1 and *hUGT1/Pxr*^−/−^ mice at 12 days after birth were treated by oral gavage with 8 mg/kg DEX for 3 days. (A) Serum bilirubin levels in untreated (Ctrl) and DEX-treated mice were evaluated in 14 day old mice (***P < 0.001, n.s., no significant difference, *t* test). (B) After DEX treatment, fold induction of *UGT1A1* gene expression in liver was determined by RT and Q-PCR analysis.

In neonatal *hUGT1* mice, UGT1A1 expression controls the levels of TSB, with significant hyperbilirubinemia developing due to limited expression of hepatic UGT1A1. We examined if glucocorticoids could regulate *UGT1A1* gene expression during the neonatal period in a PXR-dependent fashion. In hUGT1*1 mice, TSB peaks 14 days after birth and ranges from 12-15 mg/dL (Fig. 6A). Within the same litters, half of the newborn hUGT1*1 mice were treated with 8 mg/kg DEX by oral gavage, whereas the other half received just vehicle. Serum bilirubin levels were determined 48 hours after treatment. DEX treatment led to a dramatic reduction in TSB (Fig. 6A). Analysis of *UGT1A1* gene expression in hepatic tissue following DEX treatment confirmed a 40-fold induction of RNA in hUGT1*1 neonatal mice (Fig. 6B). When we examined TSB levels in response to 8 mg/kg DEX treatment in hUGT1*1/Pxr^−/−^ mice, the serum bilirubin levels did not change when compared to vehicle-treated neonatal mice (Fig. 6A). There was also no induction of hepatic UGT1A1 as determined by RT and Q-PCR analysis (Fig. 6B). Thus, DEX induces hepatic UGT1A1 leading to bilirubin metabolism through a PXR-dependent mechanism.

### PXR Represses Human *UGT1A1* Gene Expression in Neonatal *hUGT1* Mice

**Fig. 7:**
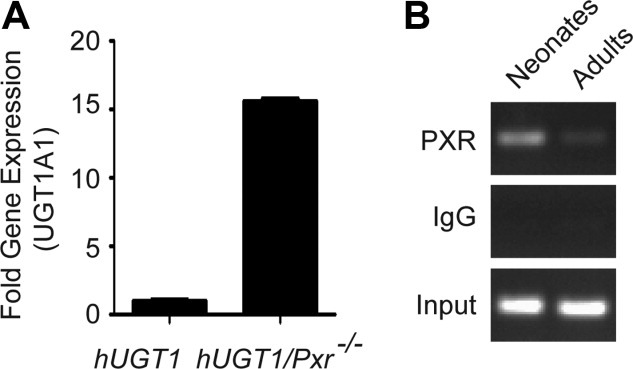
PXR represses *UGT1A1* gene expression in neonatal hUGT1*1 mice. (A) Comparison of the hepatic expression levels of the *UGT1A1* gene in *hUGT1* and *hUGT1/Pxr*^−/−^ mice 14 days after birth. (B) CHIP analysis of PXR associated with the human *UGT1A1* gene in neonatal and adult hUGT1*1 mice.

As we undertook these experiments, we also noted that TSB levels in neonatal hUGT1*1/Pxr^−/−^ peak to only 4-6 mg/dL, almost 10 mg/dL lower than observed in hUGT1*1 mice (Fig. 6A). When we examined liver *UGT1A1* gene expression at 14 days after birth, there was over a 10-fold increase in gene expression in hUGT1*1/Pxr^−/−^ mice when compared to hUGT1*1 mice (Fig. 7A). This finding indicates that nonliganded PXR during development in hUGT1*1 mice is serving to repress liver *UGT1A1* gene expression, because in PXR-deficient mice we observe induction of liver UGT1A1, which correlates with a reduction in serum bilirubin.

**Fig. 8:**
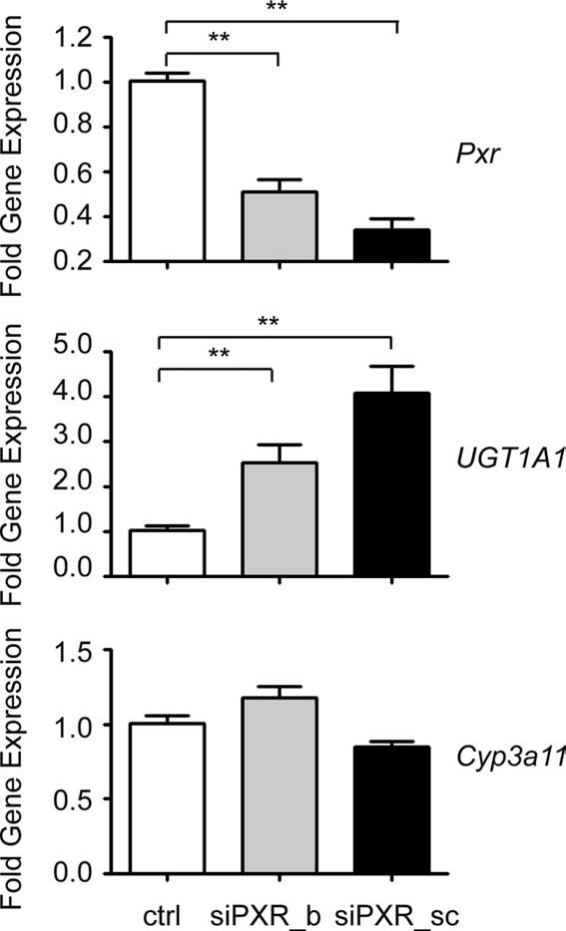
Knockdown of *PXR* and derepression of *UGT1A1* gene expression in primary hepatocytes. Hepatocytes were isolated from 14-day-old neonatal hUGT1*1 mice. Cells were transfected with mouse siRNA oligonucleotide manufactured by Bioneer (siPXR_b) and Santa Cruz Biotechnology (siPXR_sc). Forty-eight hours later, cells were harvested for RNA preparation. The relative gene expression levels of mouse *Pxr*, human *UGT1A1*, and mouse *Cyp3a11* were determined by RT and Q-PCR (**P < 0.01, *t* test).

To further examine the developmental properties of PXR on *UGT1A1* gene repression, we performed PXR CHIP analysis by using liver samples from both neonatal and adult hUGT1*1 mice. As shown in Fig. 7B, intensified PXR signals were observed in neonatal livers in comparison to adult livers. Abundant PXR binding to the *UGT1A1* gene is concordant with reduced *UGT1A1* gene expression, indicating that PXR is repressing gene expression. To examine this possibility, we isolated primary hepatocytes from 14-day-old hUGT1*1 mice and transfected them with PXR-specific siRNAs from two sources (Fig. 8). Forty-eight hours later, *UGT1A1* gene expression was quantitated by using RT and Q-PCR. The fold induction was tied to the extent of the PXR mRNA knockdown. When the PXR knockdown was 50% (siPXR_b), ≍2-fold induction of human *UGT1A1* expression was observed. When the PXR knockdown was 70%, *UGT1A1* gene induction was 4-fold. In contrast, *Cyp3a11*, another PXR target gene, showed no change. These findings confirm that transcriptional silencing of the *UGT1A1* gene by PXR occurs during neonatal development in liver tissue.

## Discussion

Employing recently developed *TgUGT1* mice with the *UGT1A* genes expressed in a *Ugt1*-null background, the function of PXR and CAR during pregnancy was investigated using reverse genetics to examine induction of the *UGT1A* genes in xenobiotic receptor-defective mice. Previous experiments with *TgUGT1* and *hUGT1* mice have shown that chemical treatment with activators of either PXR or CAR leads to induction of UGT1A1, -1A3, -1A4, -1A6, and -1A9.^14^ The mechanisms by which PXR and CAR control the induction of all these genes have not been determined, although PXR and CAR-responsive elements have been shown to play an important role in PXR/CAR binding and induction of the *UGT1A1* gene. In primary hepatocytes from *hUGT1* mice, selective treatment with 17β-estradiol, progesterone, and DEX each led to induction of the *UGT1A1* gene, with glucocorticoid treatment maximizing the induction response. In timed pregnancy experiments, each of the maternal liver-specific *UGT1A* genes was induced in *hUGT1* mice, with *UGT1A1, UGT1A6*, and *UGT1A9* gene expression being the most prominent. During pregnancy, gestational induction of the *UGT1A* genes was mostly conserved in *hUGT1/Car*^−/−^ mice but greatly diminished in *hUGT1/Pxr*^−/−^ mice, suggesting that PXR participates in a global fashion to regulate the *UGT1* locus during fetal development. The sole exception to this appears to be with *UGT1A9* gene expression, which displayed reduced expression during pregnancy in *hUGT1/Car*^−/−^ mice. Because both PXR/CAR appear to be necessary for induction of UGT1A9 during pregnancy, there may be crosstalk occurring between the two xenobiotic receptors to facilitate regulation of the *UGT1A9* gene.

The contribution of PXR toward induction of the human *UGT1* locus is not conserved with the murine *Ugt1* locus, because pregnancy has no effect on regulation of the murine *Ugt1a* genes as determined by gene expression profiling and protein accumulation. This was surprising because it has been demonstrated previously that overexpression of the human PXR in humanized PXR mice or treatment of mice with PXR ligands, such as PCN, leads to induction of the murine *Ugt1a1* gene.^3^, ^27^, ^28^ Our results indicate that PXR is the central modulator of the human *UGT1A* genes during pregnancy, but additional regulatory events specifically toward control of the human *UGT1* locus, and not the murine *Ugt1* locus, are in place during pregnancy. Because an increase in UGT1A-dependent glucuronidation occurs in humans during pregnancy, the ability to reproduce this event in transgenic mice indicates that the genetic sequence specific to the *UGT1* locus is largely responsible for directing the transcriptional program of the human *UGT1A* genes during pregnancy. Thus, the differences observed in the induction patterns between the human *UGT1A* genes and the murine *Ugt1a* genes are not the result of interspecies differences in epigenetic machinery or the cellular environment. From this result we can infer that transcriptional factors play a secondary role in dictating the differences observed in the human *UGT1* and murine *Ugt1* locus during pregnancy. Such a pattern expressing conserved genes between humans and mice has been demonstrated,^27^-^29^ reinforcing the hypothesis that differences in gene expression between species are controlled by changes in *cis*-acting transcriptional binding sequences.^21^, ^22^, ^30^

Among the UGT1A isoforms, UGT1A1 is of special physiological importance because it is the only enzyme that catalyzes the glucuronidation of bilirubin.^6^ Accumulation of bilirubin leads to benign levels of hyperbilirubinemia shortly after birth, but if bilirubin levels continue to rise, the more serious symptoms associated with bilirubin-induced neurological dysfunction (BIND) can develop.^31^, ^32^ Phenobarbital, a CAR agonist, has been used clinically for the treatment of neonatal hyperbilirubinemia in infants at risk for severe jaundice (TSB levels more than 16 mg/dL), therefore reducing the need for exchange transfusion.^33^-^35^ However, phenobarbital treatment is not effective immediately, and it diminishes the oxidative metabolism of bilirubin, increasing the risk of neurotoxic effects.^36^ Glucocorticoids have also been used to treat hyperbilirubinemia.^37^ The initial intent of glucocorticoid therapy is to help fetal lung maturation and reduce neonatal mortality in women at high risk for preterm labor before 35 gestational weeks. During these treatments, it has been observed that hyperbilirubinemia is significantly lower in the DEX-treated groups compared to untreated control groups. Our studies indicate that PXR serves as a major regulator following glucocorticoid treatment by inducing liver UGT1A1 expression, leading to reduction of hyperbilirubinemia. Identification of PXR as a key regulator of the *UGT1A1* gene during neonatal development can be exploited as a potential therapeutic target in the treatment of hyperbilirubinemia.

Analysis of TSB levels in neonatal *hUGT1* and *hUGT1/Pxr*^−/−^ mice demonstrate that PXR plays a key role in controlling serum levels during development. Neonatal *hUGT1* mice develop severe hyperbilirubinemia due to a reduction in liver *UGT1A1* gene expression.^15^ In *hUGT1/Pxr*^−/−^ mice, liver UGT1A1 is induced when compared to expression in *hUGT1* mice. The increased levels of liver UGT1A1 in *hUGT1/Pxr*^−/−^ mice lead to reduced levels of TSB. This finding indicates that in the absence of endogenous/exogenous ligands, the physiological role of PXR leads to repression of the *UGT1A1* gene during early development. It may also be an underlying regulator of the *UGT1A1* gene in newborns and responsible in part for neonatal hyperbilirubinemia. Hence, PXR acts as a repressor of *UGT1A1* expression in the absence of ligand. It is known that the repressive function of PXR works in part through the recruitment of the corepressor Silencing Mediator of Retinoid and Thyroid Hormone Receptors (SMRT).^38^, ^39^ SMRT binds to nuclear receptors in the absence of ligand and alters the chromatin structure through histone modification.^40^, ^41^ Clearly, deletion of PXR releases the repression (de-repression) allowing for spontaneous induction of *UGT1A1* gene expression. This finding may be useful in future studies to identify PXR modulators that might directly influence bilirubin homeostasis and accelerate bilirubin metabolism and clearance in children with abnormally high levels of TSB.

## Abbreviations

CAR: constitutive androstane receptor
CHIP: chromatin immunoprecipitation
ERα: estrogen receptor alpha
GD: gestational day
*hUGT1*: humanized *UGT1* mouse
PXR: pregnane X receptor
*TgUGT1*: transgenic *UGT1* mouse
TSB: total serum bilirubin
UGT: UDP glucuronosyltransferase
*UGT1*: human *UGT1* locus
*Ugt1*: murine *Ugt1* locus
