# Supplementary material for: Pregnane-X-Receptor Controls Hepatic Glucuronidation During Pregnancy and Neonatal Development in Humanized UGT1 Mice
Source: Hepatology. 2012 Jun 11;56(2):658–67. doi: 10.1002/hep.25671 (PMC3383890; doi:10.1002/hep.25671)
Supplement: Supplementary file 1 [file hep0056-0658-SD1.doc]

Supporting Table 1. Primers used for Q-PCR.

| Target | GenBank Accession No. | Orientation | Sequence (5’-3’) | Nucleotide Location |
| --- | --- | --- | --- | --- |
| UGT1A1 | NM_000463 | Sense | AACAAGGAGCTCATGGCCTCC | 412-432 |
|  |  | Anti-sense | GTTCGCAAGATTCGATGGTCG | 1056-1036 |
| UGT1A3 | NM_019093 | Sense | CAGTGGTGGATATTCTCAGTC | 731-751 |
|  |  | Anti-sense | CCATGTTCTCCAGAAGCATTA | 905-885 |
| UGT1A4 | AY_435139.1 | Sense | ACGCTGGGCTACACTCAAGG | 277-296 |
|  |  | Anti-sense | TCATTATGCAGTAGCTCCACACAA | 404-381 |
| UGT1A6 | AY_435141.1 | Sense | CTTTTCACAGACCCAGCCTTAC | 439-460 |
|  |  | Anti-sense | TATCCACATCTCTCTTGAGGACAG | 727-704 |
| UGT1A9 | AY_435144.1 | Sense | GAGGAACATTTATTATGCCACCG | 643-665 |
|  |  | Anti-sense | GCAACAACCAAATTGATGTGTG | 760-739 |
| Ugt1a1 | NM_201645.1 | Sense | GTGTGTTCGGTCCCTATGCAT | 102-122 |
|  |  | Anti-sense | CGGCATTGTGCAGCAGGTGGG | 452-432 |
| Ugt1a6 | NM_201410.1 | Sense | CCAAGCCCTGTGTCCTATGT | 659-678 |
|  |  | Anti-sense | TTGAGGAGGTCTGAGGCAAT | 816-797 |
| Cyp3a11 | NM_007818.3 | Sense | CCGATGTTCTTAGACACTGCC | 1228-1248 |
|  |  | Anti-sense | CTCAATGGTGTGTATATCCCC | 1650-1630 |
| Mouse CPH | XM_913899.3 | Sense | ATGGTCAACCCCACCGTGT | 48-66 |
|  |  | Anti-sense | TTCTTGCTGTCTTTGGAACTTTGTC | 146-126 |
| Pxr | NM_010936.3 | Sense | CCTTTGACACAACTTTCTCCC | 808-826 |
|  |  | Anti-sense | CAGGGTCTTCCAACAGTGAG | 915-896 |
